# Kinetics of Biomarkers for Therapeutic Assessment in Swiss Mice Infected with a Virulent *Trypanosoma cruzi* Strain

**DOI:** 10.3390/pathogens15010107

**Published:** 2026-01-19

**Authors:** María Fernanda Alves-Rosa, Doriana Dorta, Alexa Prescilla-Ledezma, Jafeth Carrasco, Leighanne Bonner, Jon J. Tamayo, Michelle G. Ng, Adelenis Vega, Melany Morales, Davis Beltran, Rosa De Jesús, Carmenza Spadafora

**Affiliations:** 1 Division of Human Health and Diseases, Instituto de Investigaciones Científicas y Servicios de Alta Tecnología (INDICASAT AIP), Panama 0843, Panama; mariaalves@indicasat.org.pa (M.F.A.-R.); lianmarie26@gmail.com (L.B.); jonj.tamayo23@gmail.com (J.J.T.); mng@indicasat.org.pa (M.G.N.); adelenis.vega03@gmail.com (A.V.);; 2 Sistema Nacional de Investigación (SNI), Secretaría Nacional de Ciencia Tecnología e Innovación (SENACYT), Panama 0843, Panamardejesus@indicasat.org.pa (R.D.J.); 3 Facultad de Medicina, Departamento de Microbiología Humana, Universidad de Panamá, Panama 0801, Panama; 4 Medical Microspectroscopy Research Group, Department of Experimental Medical Science, Faculty of Medicine, Lund University, 22184 Lund, Sweden; 5 Escuela de Biotecnología, Facultad de Ciencias de la Salud, Universidad Latina de Panamá, Panama 0823, Panama; 6 Facultad de Ciencias Naturales, Exactas y Tecnología, Departamento de Genética y Biología Molecular, Universidad de Panamá, Panama 0801, Panama; 7 Flow Cytometry Core, GMIHS, Gorgas Memorial Institute of Health Sciences, Panama 0816, Panama; 8 Bioterio, Instituto de Investigaciones Científicas y Servicios de Alta Tecnología (INDICASAT AIP), Panama 0843, Panama

**Keywords:** *T. cruzi*, chagas disease, Swiss mice, animal model

## Abstract

Chagas disease (CD), caused by *Trypanosoma cruzi*, is a neglected tropical illness affecting 6–8 million people in Latin America. Reaching scholarly consensus on the host response to *T. cruzi* infection remains a significant challenge, primarily due to substantial heterogeneity in outcomes driven by both the choice of animal model and the infecting parasite’s discrete typing unit (DTU). This variability complicates the evaluation and comparison of new therapeutic compounds against existing drugs, namely benznidazole and nifurtimox. This study provides a comprehensive, kinetic, multifaceted characterization of the acute infection using the highly virulent *T. cruzi* Y strain (TcII) in outbred Swiss mice. Here, crucial infection parameters are presented, including the optimal infective dose, the parasitemia dynamics, tissue damage markers, hematological profiles, cytokine production (Th1/Th2/Th17/Th22), and molecular parasite identification in target organs (heart, colon, esophagus, spleen, and liver) across the span of the infection. The novelty of this study lies in the kinetic integration of these parameters within a defined model; rather than presenting isolated data points, we demonstrate how the biochemical, physiological, and clinical signs and immunological responses, with the resulting organ involvement, evolve and interact over time. To complete the report, a necropsy evaluation was performed at the end of the acute, fatal infection, and it is presented here. This study fulfills a long-standing recommendation from diverse drug discovery groups for the creation of a definitive reference model to standardize preclinical testing for anti-Chagasic agents.

## 1. Introduction

*Trypanosoma cruzi* is a parasitic protozoan that causes Chagas disease (CD), also known as “American trypanosomiasis”. According to the World Health Organization (WHO), it affects between 6 and 8 million people, with an additional 70 million at risk, and it is the most prevalent neglected tropical disease in Latin America. The most vulnerable populations are those with limited financial resources, as the disease is closely linked to precarious housing conditions, such as cracked walls and thatched roofs, that facilitate the proliferation of the triatomine vector. Research on CD is primarily concentrated in high-incidence countries, including Brazil, Argentina, and Panama [1]; however, it receives limited attention from the global scientific community.

A striking feature of *T. cruzi* is its heterogeneity in terms of biological properties. Currently, six distinct lineages are classified, based on biochemical and molecular markers, into discrete typing units (TcI-VI) [2], which vary their geographic occurrence, host specificity, and pathogenicity [3,4]. Differences in growth rates, infectivity, tissue tropism, antigenic composition, virulence, and morbidity have been reported among parasite isolates in animal models, along with susceptibility to immune sera and chemotherapeutic drugs [5,6,7,8,9]. These variations make it difficult to reach consensus on host–pathogen behavior, parasite-associated pathology, and, consequently, the development of new treatment strategies.

Up to one-third of chronic disease patients develop fatal heart and digestive problems [10], causing around 10,000 deaths annually. The current treatments, nifurtimox and benznidazole, have been used for over 50 years and are associated with severe side effects, prolonged treatment durations, and low efficacy in the chronic phase [11], making patient adherence challenging. Despite some advances in drug development, no effective alternatives have yet been identified, highlighting the urgent need for new therapeutic molecules that are more specific and have fewer adverse effects. The introduction of new therapies would particularly benefit socioeconomically, ethnically, and geographically marginalized groups, contributing to a reduction in health disparities. Advancing knowledge on CD and developing novel therapeutic alternatives would enhance prevention and treatment strategies. The development of a new drug begins with an in vitro evaluation to determine its effects and therapeutic window. Once efficacy has been established, the research proceeds to in vivo studies, typically using rodent models.

A crucial step in carrying out successful in vivo drug testing is selecting the pathogen strain. A highly virulent, potent strain presents a challenging scenario for any therapeutic molecule. If a new drug proves effective against such a strain, it is more likely to be effective against less virulent strains of the same pathogen., The Y strain of *T. cruzi* is an excellent choice for these studies, as it belongs to the second most spread DTU (TcII) in the Americas and is highly virulent due to its increased resistance to the lectin and C2 complement receptors, surpassing even the Colombian strain in this regard. When murine acute infection models are used, the Y strain induces as high a virulence as the Colombian strain [12,13,14,15].

Additionally, selecting an appropriate murine host is essential. Inbred and outbred mice differ in genetic diversity and, consequently, in the degree of experimental variability. As humans are genetically heterogeneous, studies using outbred mice more accurately reflect the spectrum of immunological and physiological responses observed in patients infected with the pathogen [16]. The benefits of using the genetically diverse Swiss Webster mice for preclinical studies have been well documented. Various authors have reported on how genetic human immunity responses, from infections studies to vaccine response analysis and inflammation assessments, are better reflected in outbred mice and even over C57BL/6 [17,18,19,20,21]. Swiss mice are outbred and sensitive to CD but more robust to infection with *Trypanosoma cruzi* than other inbred lines such as BALBc. Using *T. cruzi* clonal populations on three inbred mic lineages and one outbred mice lineage, Andrade et al. observed the same tissue distribution of the parasite in the two inbred animals, probably due to them having the same H-2 haplotype [22], highlighting the importance of choosing an outbred host to better mimic real live infections. Thus, Swiss mice, with their more diverse genetic background than inbred lines, are optimal hosts to test drugs against *T. cruzi* [23,24]. This combination of the *T. cruzi* strain and an experimental animal host was agreed upon among several CD drug discovery laboratories in Latin America as a benchmark for comparing results and identifying promising compounds for further development [25].

This study provides a multifaceted characterization of acute *T. cruzi* Y infection in Swiss mice. At the end of this study, a list of parameters are presented that can be used to monitor the effect of any therapeutic intervention, such as an experimental compound, to help inform Go/No-Go decisions in drug discovery while avoiding analyzing irrelevant variables.

Important parameters were analyzed: the appropriate number of trypomastigotes required to achieve an early peak of parasitemia without causing host mortality; biochemical parameters such as tissue damage markers; hematological profiles; a study of Th1/Th2, as well as Th17 and Th22, cytokine production at significant time points of the infection; the molecular identification of the presence of the parasite in organs considered potential targets of the pathogen, namely heart, colon, esophagus, spleen, and liver; and a histological evaluation of the said tissues to assess the localization of the parasite, damage, and inflammation. The results provide a comprehensive set of parameters that enable the identification of critical parameters for evaluating any compound considered a potential anti-Chagasic drug.

## 2. Materials and Methods

***T. cruzi* Y Strain** **.**

The *T. cruzi* Y strain was provided by Dr. André Talvani from Laboratório de Imunobiologia da Inflamação, Departamento de Ciências Biológicas, Instituto de Ciências Exatas e Biológicas (ICEB), Universidade Federal de Ouro Preto (Minas Gerais, Brazil). To maintain the strain virulence, constant mice passages were performed weekly. For experimentation, the method below was used.

**Sample preparation for in vivo *T. cruzi* infection** **.**

Vero cells were maintained in RPMI 1640 medium (RPMI) supplemented with 10% fetal bovine serum (FBS) and streptomycin. Upon reaching 70–80% confluence, the cells were inoculated with 150 µL of whole blood collected via submandibular puncture from infected mice, and the culture was incubated at 37 °C in a 5% CO_2_ atmosphere for 24 h to facilitate parasite adhesion and internalization. Subsequently, non-internalized parasites were removed via washing with RPMI 1640. To ensure sufficient parasite density for formal experiments, they underwent three successive passages in Vero cells. After three passages in Vero cells to amplify the parasites, they were used for the formal experiments, as described below.

Five days post-infection (d.p.i.), extracellular trypomastigotes were isolated from the culture supernatant, The supernatant was collected and centrifuged at 1500 rpm for 10 min. For quantification, the parasites were immobilized in RPMI containing 1% formaldehyde and counted using a Neubauer chamber. The resulting inoculum was adjusted to a concentration of 10^4^ trypomastigotes per 200 μL of sterile RPMI. Additionally, the *T. cruzi* strain was maintained in Swiss mice through weekly serial passages using an inoculum of 4–5 × 10^2^ blood-form trypomastigotes.

**Experimental *T. cruzi* infection** **.**

Swiss female mice were used, following the consensus-based experimental protocol for anti-Chagas drug testing described by Romanha et al. [25]. Pathogen-free animals were obtained from and housed at INDICASAT AIP. Mice were inoculated intraperitoneally (i.p.) either with 200 μL of RPMI alone (control) or RPMI containing 5 × 10^4^ viable trypomastigotes of *T. cruzi* Y, corresponding to the *T. cruzi* lineage II [3].

**Animals** **.**

Swiss Webster mice, aged 4–8 weeks old, (25–30 g) were used, following the standardized experimental model for anti-Chagas drug testing described by Romanha et al. [25]. Pathogen-free animals were obtained from and housed at the Instituto de Investigaciones Científicas y Servicios de Alta Tecnología de Panamá (INDICASAT AIP) Animal Facility in standard clear plastic cages appended to a ventilated rack (Model LN63FESU, Tecniplast, Buguggiate, Italy) with free access to water and food, a light/dark cycle of 12/12 h, and controlled temperature. The animals were housed in groups of no more than five per cage. Mice were acclimated to the testing room for at least 1 h before the beginning of the experiments, which were conducted during the light cycle, i.e., between 7:00 a.m and 7:00 p.m.

All animals originated from the same supplier. The production system used was based on the Robertson system, which allows for genetic variability according to the established standards for colonies with fewer than 1000 breeders. Three pairs per breeder block were used and there were eight established blocks, following the established breeder replacement schedule for the system [26].

**Experimental Design** **.**

**Experiment I. Kinetics of Parasitemia and Animal Responses** **.**

A total of 10 female Swiss mice were used in this study. The animals were individually identified via tail markings to facilitate longitudinal monitoring.

**Longitudinal Clinical Assessment and Blood Sampling** **.**

On days 1, 4–11, and 13–17 post-treatment, *T. cruzi*-infected animals were clinically assessed for physical appearance, behavior, body temperature, and body weight. Body weight and body temperature were recorded at each time point using a calibrated digital balance and a digital thermometer, respectively. Food and water intake were recorded weekly. Blood was subsequently collected via the tail vein to evaluate changes in hematological parameters and parasitemia, as described below.

On days 4–11, and 13–14 p.i., mice were bled via the tail vein. All blood collections were performed within the same time window and using the same sampling route to minimize variability associated with circadian rhythm or collection technique.

Baseline hematological parameters were determined by collecting 20 µL of blood from the tail vein of each animal, using tubes containing EDTA K2 to prevent coagulation.

Following baseline blood collection, mice were inoculated intraperitoneally (i.p.) with 100 μL of RPMI containing 5 × 10^4^ viable trypomastigotes of *T. cruzi* Y, corresponding to *T. cruzi* lineage II [27].

In total, 15 µL of blood was analyzed using the Mythic 18 VET analyzer (Orphee, Switzerland) with the M-Pack kit. The following hematological parameters were evaluated: white blood cell count (WBC), lymphocyte percentage, absolute lymphocyte count, monocyte percentage, absolute monocyte count, granulocyte percentage, absolute granulocyte count, red blood cell count (RBC), hemoglobin concentration, hematocrit (HCT), mean corpuscular hemoglobin (MCH), mean corpuscular hemoglobin concentration (MCHC), red cell distribution width (RDW), platelet count, mean platelet volume (MPV) and plateletcrit (PCT).

**Parasitemia Determination** **.**

For parasitemia assessment, 5 µL of the collected blood was placed on a microscope slide and covered with a 24 × 24 mm^2^ coverslip (total area: 576 mm^2^). Samples were examined using a light microscope at 40× magnification with an ocular Field Number (FN) of 20.

The field of view (FOV) diameter was calculated as

FOV (mm) = FN/40 = 0.50 mm

Assuming a circular field, the area per microscopic field was calculated as

Field area = *n* (0.50/2)^2^ = 0.196 mm^2^

A total of 50 microscopic fields were examined; thus, the area analyzed was 9.8 mm^2^, representing 1.7% of the total coverslip area.

Parasite counts obtained in the 50 fields were extrapolated to the total coverslip area containing 5 µL of blood using the following equation:

Parasites per coverslip = parasites counted in 50 fields/9.8 × 576

Parasite concentration was then expressed as parasites per milliliter of blood as follows:

Parasites per mL = parasites per coverslip × 1000 µL/5 µL

**Biochemical Markers I** **.**

Baseline and final biochemical markers were determined by collecting 150 µL of blood via the submandibular route before the mice were inoculated intraperitoneally (i.p.) with 100 μL of RPMI containing 5 × 10^4^ viable *T. cruzi* Y trypomastigotes and again at day 14 p.i. Samples were collected into MiniCollect^®^ TUBE 0.5/0.8 mL CAT Serum Sep Clot Activator tubes (Greiner Bio-One GmbH, Kremsmünster, Austria) for serum preparation for biochemical analysis. After collection, samples were centrifuged at 3500 rpm for 10 min, and the serum was collected and stored at 4 °C until processed.

Biochemical analysis was performed using a FUJIFILM dry chemistry analyzer. The following parameters were evaluated: ALT (alanine aminotransferase), a marker of liver damage; LDH (lactate dehydrogenase), an indicator of tissue damage, particularly in the heart or liver; and CPK (creatine phosphokinase), a marker of muscle, cardiac, or brain injury.

**Experimental Endpoint** **.**

All animals were euthanized at the end of the experiment. Anesthesia was induced via the inhalation of 10% isoflurane in a glass chamber containing a cotton ball soaked with the anesthetic. Direct contact with the anesthetic was prevented using a protective tube. After 3–5 min of exposure, heart rate and pedal reflex were assessed. Once the pedal reflex was absent, euthanasia was performed by opening the thoracic cavity.

**Experiment II. Kinetics of Tissue Infection, Biochemical markers II, and Cytokine Production** **.**

Forty female Swiss Webster mice (Taconic Biosciences, Rensselaer, NY, USA) were used in this experimental procedure. Animals were individually identified by tail marking to allow for follow-up. Mouse cages were randomly assigned to the experimental groups. Twenty mice were inoculated intraperitoneally (i.p.) with RPMI alone (control group), and twenty mice were inoculated i.p. with RPMI containing 5 × 10^4^ viable *Trypanosoma cruzi* trypomastigotes (infected group). Animals were individually identified by tail marking to allow for longitudinal follow-up.

On days 4, 7, 11, and 14, five randomly selected control animals and five randomly selected *T. cruzi*-infected mice were anesthetized via the inhalation of 5% isoflurane, as described previously, and subjected to cardiac puncture to collect 1 mL of blood for biochemical and immunological assays. The samples were collected into MiniCollect^®^ TUBE 0.5/0.8 mL CAT Serum Sep Clot Activator tubes (Greiner Bio-One) for serum preparation. Serum samples were centrifuged at 3500 rpm for 10 min, aliquoted, and stored at 4 °C until processed.

**Cytokine Detection** **.**

Serum Th1/Th2 profiles (IFN-γ, IL-5, TNF-α, IL-2, IL-6, IL-4, IL-10, IL-13) were quantified from control and *T. cruzi*-infected mice using the LegendPlex™ mouse Th1/Th2 Panel CBA kit according to the manufacturer’s instructions (Biolegend, San Diego, CA, USA) and analyzed via a BD LSRFortessa™ Flow Cytometer and the Data Analysis Software Suite for LEGENDplex, versión 2025-05-01 (Qognit Inc, San Carlos, CA, USA).

IL-22 and IL-17A/F levels were measured using ELISA kits (BioLegend), following the standard protocols provided by the manufacturer. The plate was read at 450 nm using a Synergy HT tool sourced from Biotek (Santa Clara, CA, USA).

**Experimental Endpoint, Necropsy, and Tissue Collection** **.**

On days 4, 7, 11, and 14, the randomly selected control animals and five *T. cruzi*-infected mice were euthanized by opening the thoracic cavity, as previously described.

A macroscopic inspection of the organs was performed, and photographic images were acquired for comparison between control and infected animals. The heart, spleen, liver, esophagus, and colon were collected from each animal; immediately placed into pre-chilled tubes on ice; and washed with sterile saline at 4 °C. Organs were then weighed, and their weights were expressed as a percentage of the total body weight of each animal.

Tissue fragments were processed for total DNA extraction according to the manufacturer’s instructions, while the remaining tissue was fixed in buffered formalin (10%) for further histopathological analysis (not presented here) or stored at −80 °C for subsequent molecular assays.

**Conventional Polymerase Chain Reaction (cPCR)** **.**

Genomic DNA was extracted using the commercial NucleoSpin Tissue kit (Macherey-Nagel, Düren, Germany), following the manufacturer’s instructions, albeit with a slight modification. This modification included an additional wash step, followed by a 2 min drying step. DNA quantification was performed using a nanodrop spectrophotometer (Thermosfisher, Waltham, MA, USA). Only samples with a A260/A280 ratio between 1.8 and 2 were further used. cPCR was performed using the primers Cruzi 1 and Cruzi 2, designed to detect satellite DNA repeats (Table 1) [27].

For the PCR, 10 uM of each primer and 10 ng of DNA were used. A no-template reaction was used as a negative control. DNA from *T. cruzi* epimastigotes was used as a positive control.

cPCR was performed using a thermocycler (Bio-Rad, Hercules, CA, USA) under the following temperature conditions: an initial step at 98 °C for 30 s, followed by 24 cycles of 10 s at 98 °C, 15 s at 58 °C, and 20 s at 72 °C. Finally, a 10 min extension at 72 °C was added. Amplicons were run on a 3% agarose gel electrophoresis and visualized under ultraviolet light.

**Animal Welfare Assessment** **.**

Animal welfare was assessed throughout this study, i.e., before, during, and after the experimental procedures [25,28,29,30,31]. Clinical indicators of severe illness were carefully monitored and documented. The protocol used in these experiments was approved by the INDICASAT IACUC with No. CICUA 23-002.

**Statistics** **.**

Statistical analysis was performed in this study using GraphPad Prism (version 9.0.0, GraphPad Software, San Diego, CA, USA). A parametric test method was used for comparing normally distributed data. Data were presented as Mean ± SD. Comparisons between two groups were conducted using the *t*-test (unpaired test). Comparisons among three or more groups were performed using one-way ANOVA, followed by Bonferroni’s test (two-tailed). *p* < 0.05 was considered statistically significant. Differences in sample size (*n*) between parameters evaluated at the same time points resulted from the exclusion of outliers identified using the ROUT method (Q = 1%) through Prism software.

## 3. Results

### 3.1. Parasite Dynamics and Burden Evaluation

**Figure 1:**
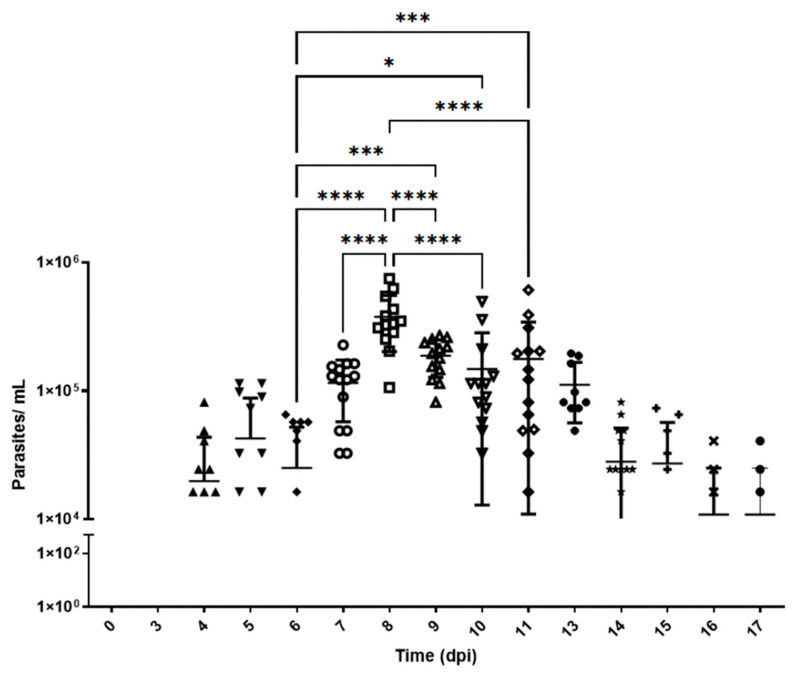
Parasitemia levels in Swiss mice infected with *T. cruzi* strain Y. Parasitemia was evaluated for 17 days. The number of parasites per milliliter (log_10_ scale) was expressed in terms of mean and standard deviation. d.p.i.: days post-infection. *, ***, and **** represent significant differences at *p* < 0.005, *p* < 0.001, and *p* < 0.0001, respectively. ANOVA was performed, followed by Bonferroni’s test (two-tailed). The data correspond to two independent experiments. Different shapes represent different dates.

Based on previously collected data (Supplementary Information S1), 4-week-old mice were intraperitoneally inoculated with 5 × 10^4^ parasites. Blood samples were collected from the mice before infection and at 4, 5, 7, 8, 9, 14, and 17 d.p.i. Figure 1 shows the parasitemia dynamics in mice infected with the Y strain of *T. cruzi*. Trypomastigotes are detectable in the blood from day 4 p.i., followed by an exponential increase that peaks on day 8 p.i., with approximately 4 × 10^5^ parasites/mL. From this point on, a gradual decline is observed until day 17 p.i., at which moment the experiment reaches its endpoint. Day 8 p.i. corresponds to the peak of parasitemia. All animals survived until the endpoint of the experiment.

The area under the curve (AUC) was calculated from the parasitemia data. This allowed for quantifying the total parasite burden in blood over time [32]. The total AUC value was 1.45 × 10^6^ parasites/day/mL ± 2.66 × 10^5^ (95% CI 9.29 × 10^5^–1.98 × 10^6^).

The rates of parasite replication and disappearance from the circulating blood were calculated using the slopes of the parasitemia curves between days 0 and 8 and between days 8 and 17, respectively. The rate of parasitemia increase during the expansion phase (0 to 8th day) was estimated at 7.72 × 10^4^ ± 1.97 × 10^4^ parasites/mL/day. Meanwhile, the parasite clearance rate during the resolution phase (9th to 17th day) was lower, i.e., 2.3 × 10^3^ ± 5.4 × 10^3^ parasites/mL/day.

### 3.2. Physiology, Behavior, and Systematic Pathology

**Figure 2:**
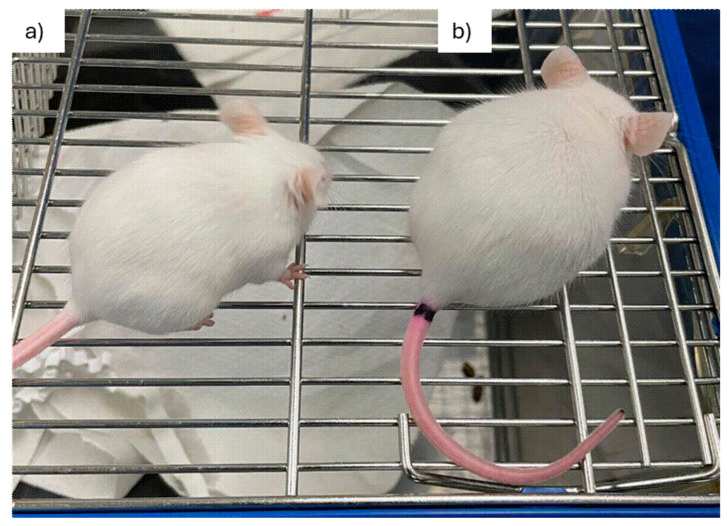
Clinical manifestations of acute infection in Swiss mice infected with *T. cruzi* strain Y. (**a**) An uninfected mouse and (**b**) a *T. cruzi* strain Y-infected mouse at 12 d.p.i., exhibiting hunched posture and piloerection. d.p.i.: days post-infection.

Throughout the first 11 days p.i., infected mice exhibited normal mobility and showed no signs of piloerection or hunching. However, piloerection was detected on day 12 p.i. in most animals (Figure 2). By day 13 p.i., this symptom had become generalized and was accompanied by hunching and reduced mobility. The control and infected mice had the same survival rate up to day 17, when the experiment ended.

Water and food consumption were measured at the onset of the experiment, on day 7 p.i., and again on day 14 p.i. As shown in Table 2, no significant differences were observed between infected and control groups in either water or food intake. This was true despite the onset of clinical symptoms, such as reduced mobility and physical deterioration, in infected animals.

To assess the physiological impact of *T. cruzi* Y infection, body temperature and weight were measured on days 0 (pre-infection), 8 (peak parasitemia), and 14 p.i.

**Figure 3:**
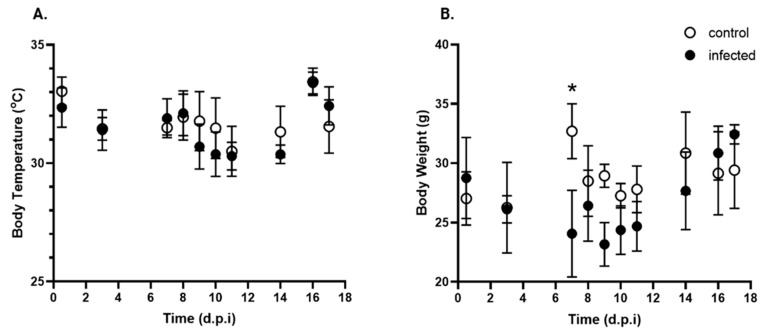
Kinetics of body temperature and body weight over time post-infection with *T. cruzi*. (**A**). Body temperature (°C) and (**B**). Body weight (g) of control (open circle) and *T. cruzi* strain Y-infected (black circle) mice were expressed as mean ± standard deviation. d.p.i.: days post-infection. * *p* < 0.05. Data were analyzed via ANOVA, followed by the Bonferroni test (two-tailed); *n* = 10 mice/time point.

Figure 3A shows no significant differences in body temperature at different time points or between the treated and control groups. Regarding body weight (Figure 3B), infected mice exhibited a significant decrease at 7 d.p.i. After this decrease, the animals appeared to recover, maintaining a similar weight for the remainder of the experiment.

### 3.3. Tissue Damage

**Figure 4:**
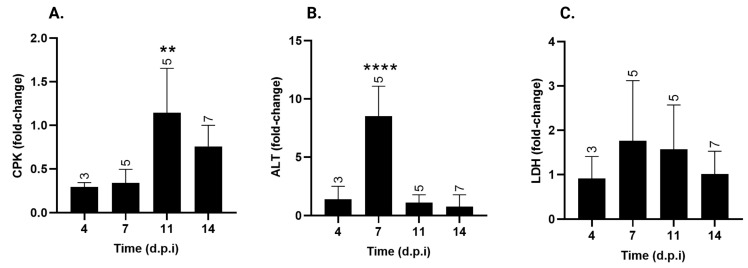
Serum creatine phosphokinase (CPK), alanine aminotransferase (ALT) and lactate dehydrogenase (LDH) levels over time post-infection with *T. cruzi* strain Y. (**A**) CPK, (**B**) ALT, and (**C**) LDH serum levels in *T. cruzi* Y-infected mice were expressed as fold-change over uninfected animals ± standard deviation. d.p.i.: days post-infection. ** *p* < 0.01; **** *p*< 0.0001. Data were analyzed via ANOVA, followed by Bonferroni’s two-tailed test. The numbers above each bar indicate the number of animals per group. The data presented here take into account biochemical markers from experiments I and II.

Biochemical parameters were evaluated to identify potential alterations associated with *T. cruzi*-induced tissue damage. CPK activity (Figure 4A), a biomarker of cardiac damage, showed a significant fold increase on day 11 p.i., following the peak of parasitemia. By day 14 p.i., CPK levels remained elevated compared to days 4 and 7 p.i., although the differences were not statistically significant. CPK levels at 4, 7, 11, and 14 d.p.i. had increased 0.3 ± 0.04-fold, 0.3 ± 0.15-fold, 1.1 ± 0.5-fold **, and 0.8 ± 0.24-fold, respectively (** *p* < 0.01 vs. 4 and 7 d.p.i).

ALT, a marker of liver damage, showed a significant increase at 7 d.p.i., one day before the peak of parasitemia (Figure 4B). At this time point, ALT levels exhibited an 8.5 ± 2.58-fold increase *** (*p* < 0.001), with no significant changes observed at the other time points.

Contrary to the other two factors, LDH serum levels (Figure 4C) remained unchanged and similar to those of non-infected mice throughout the experiment.

### 3.4. Hematological Profile

Hematological evaluations were performed to monitor alterations in blood parameters that could be associated with disease progression.

#### 3.4.1. Peripheral White Cell Counts

**Figure 5:**
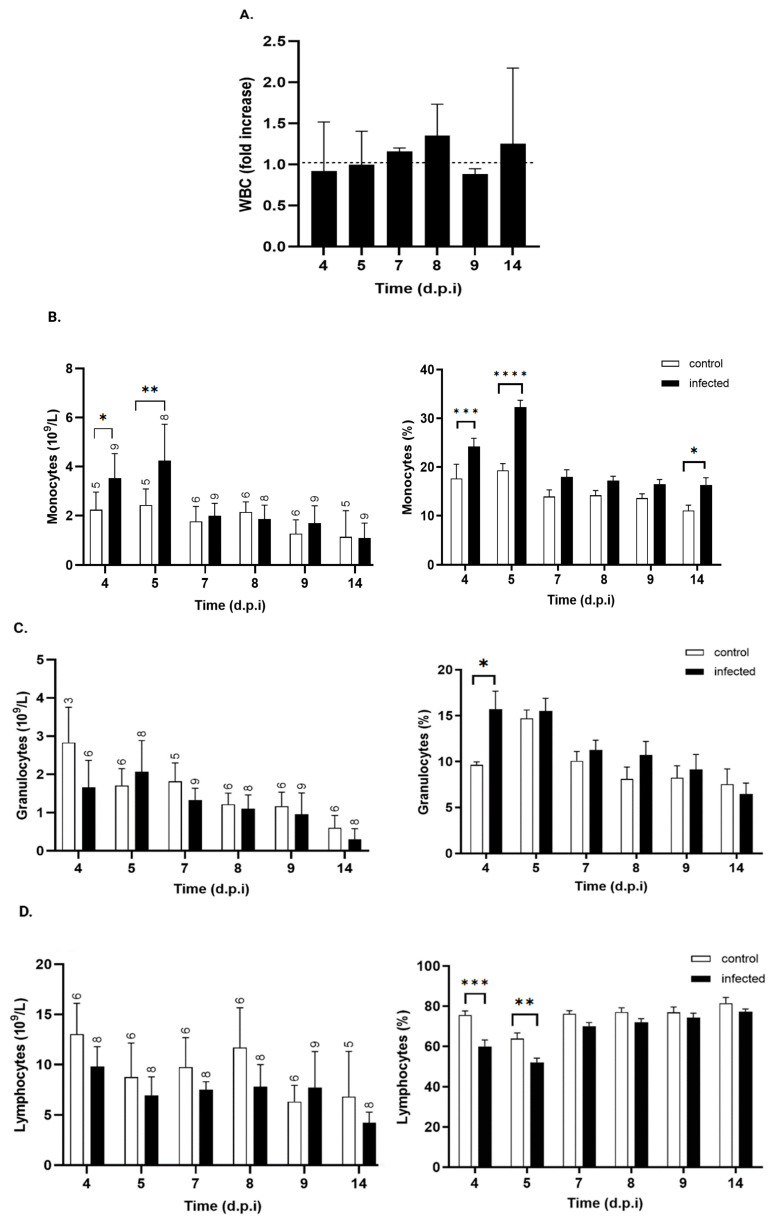
Kinetics of total and differential white blood cell counts in control and *T. cruzi* strain Y-infected mice. (**A**). Total white blood cell count (WBC) expressed as fold increase over control mice at each time point (black bars). Absolute (**right**) and relative (**left**) counts of (**B**) monocytes, (**C**) granulocytes, and (**D**) lymphocytes from control (white bars) and *T. cruzi* strain Y-infected mice (black bars). Values are expressed as mean ± standard deviation. d.p.i.: days post-infection. * *p* < 0.05; ** *p* < 0.01; *** *p* < 0.001; **** *p* < 0.0001. Data were analyzed through ANOVA, followed by Bonferroni’s two-tailed test. The numbers above each bar indicate the number of animals per group.

Total leukocytes showed no significant changes (Figure 5A). However, differential analysis revealed an increase in the absolute monocyte count in infected mice at days 4, 5, and 14 p.i. (Figure 5B). This increase was transient, with values returning to baseline by day 7 p.i.

During the same period, no significant changes were observed in the absolute counts of granulocytes (Figure 5C) or lymphocytes (Figure 5D). The relative distributions of leukocyte subsets also showed significant increases in the percentage of monocytes at days 4 and 5 p.i. (Figure 5B) and a higher granulocyte percentage in infected mice by day 4 p.i. (Figure 5C), suggestive of early innate immune activation. This increase was short-lived, with granulocyte percentages returning to baseline levels by day 5 p.i. Conversely, the percentage of lymphocytes in infected mice decreased significantly on days 4 and 5 (Figure 5D), returning to control values by day 7 p.i. From that point onward, lymphocyte proportions remained stable, with no significant differences between groups recorded up to day 14 p.i.

#### 3.4.2. Erythrocyte Parameters

**Figure 6:**
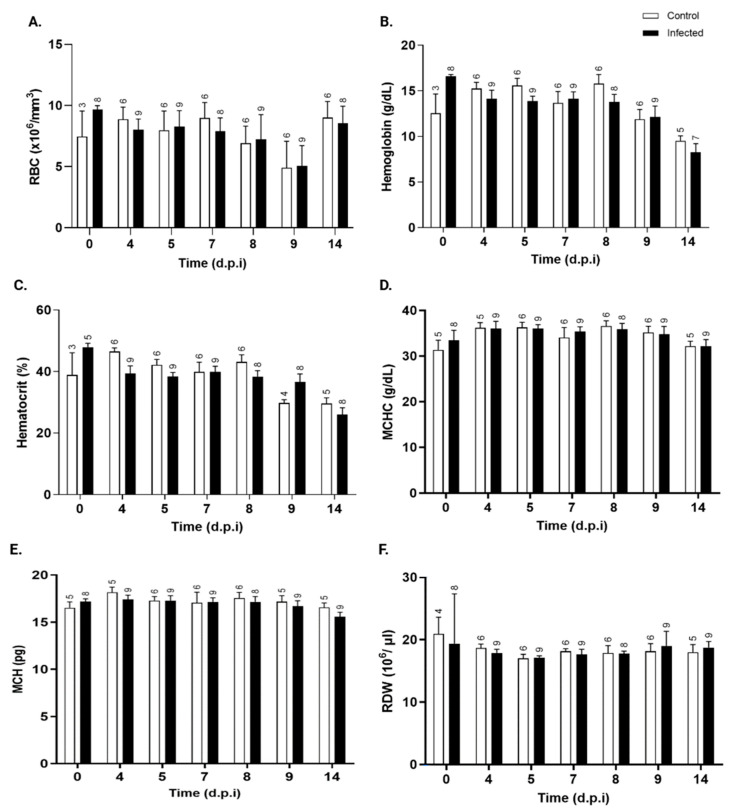
Kinetics of erythrocyte parameters in control and *T. cruzi* Y-infected mice. (**A**) total red blood cells (×10^6^/mm^3^), (**B**) hemoglobin (g/dL), (**C**) hematocrit (%), (**D**) mean corpuscular hemoglobin concentration (MCHC, g/dL), (**E**) mean corpuscular hemoglobin (MCH, pg), and (**F**) red cell distribution width (RDW, %) in peripheral blood in control (white bars) and *T. cruzi* strain Y-infected Swiss mice (black bars) were expressed as mean ± standard deviation and analyzed through ANOVA, followed by Bonferroni’s two-tailed test. The numbers above each bar indicate the number of animals per group. d.p.i.: days post-infection.

Erythrocyte parameters did not differ significantly between the *T. cruzi*-infected and control groups. Total red blood cell (RBC), hemoglobin, hematocrit, mean corpuscular hemoglobin concentration (MCHC), mean corpuscular hemoglobin (MCH), and red cell distribution width (RDW) values (Figure 6A–F, respectively) remained stable and similar between the groups throughout the infection period.

#### 3.4.3. Platelet Parameters

**Figure 7:**
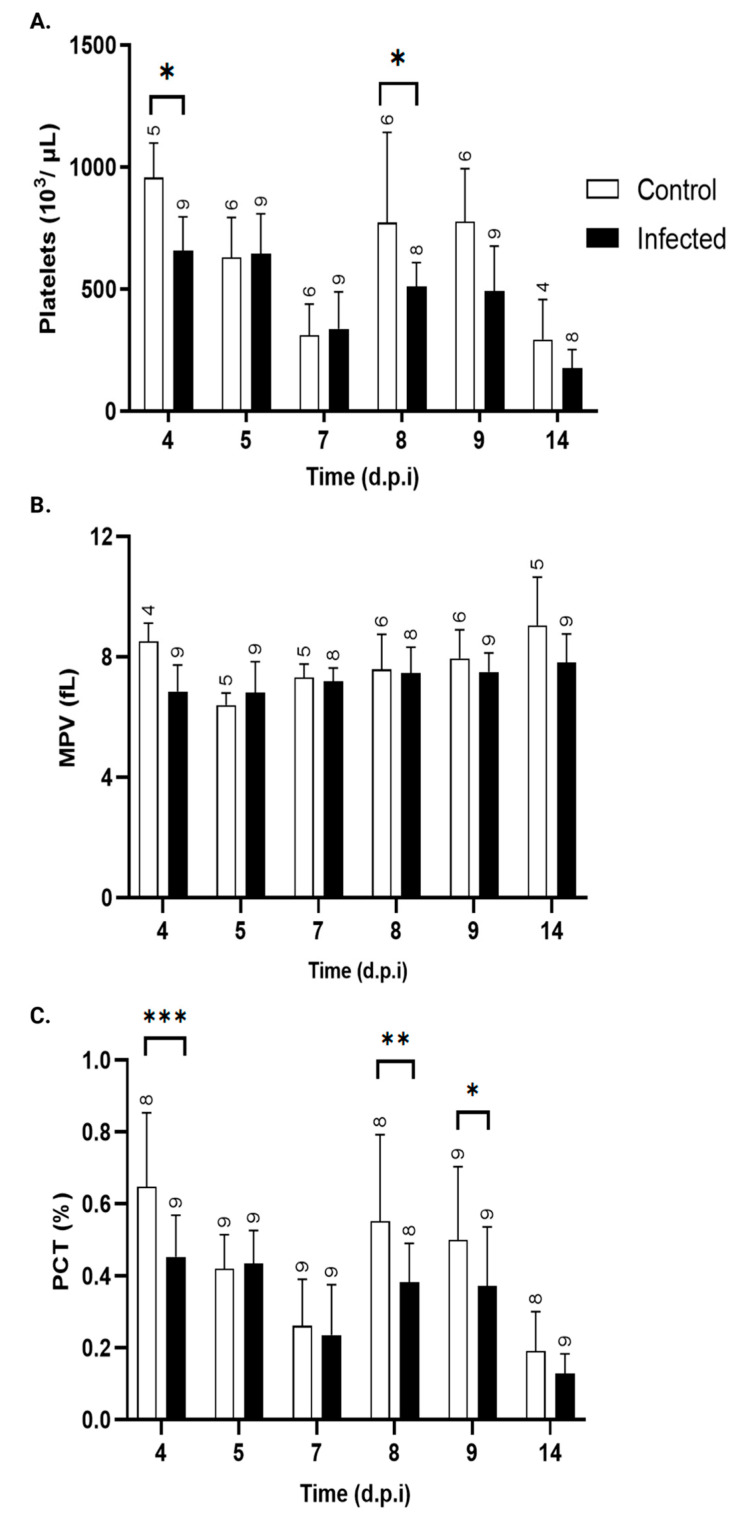
Platelet parameters. (**A**) platelet count (10^3^/μL), (**B**) plateletcrit (PCT, %), and (**C**) mean platelet volume (MPV, fL) in peripheral blood of control (white bars) and *T. cruzi* strain Y-infected Swiss mice (black bars). Values are expressed as mean ± standard deviation. d.p.i.: days post-infection. * *p* < 0.05, ** *p* < 0.01, and *** *p* < 0.001, ANOVA, followed by a two-tailed Bonferroni test, was used to analyze significance. The numbers above each bar indicate the number of animals per group.

Figure 7A illustrates the changes in platelet counts over the post-infection period. On day 4, when parasites begin to appear in the bloodstream, mice infected with *T. cruzi* showed a significant decrease in platelet number. However, by day 5 and 7, this difference was no longer statistically significant. From day 8 p.i. onward, platelet counts progressively increased, reaching significance on day 9. These findings are supported by kinetic analysis of the plateletcrit (Figure 7B), which shows a trend consistent with the reduction in the platelet count. The mean platelet volume (MPV) values remained unchanged throughout the study period (Figure 7C).

### 3.5. Immune Response (Cytokines)

Serum cytokine kinetics are depicted in Table 3. Cytokines of the Th1/Th2 profile (IFN-γ, TNF-α, IL2, IL-6, and IL-10), strict Th2 (IL-5 and IL-4), and Th17 (IL17/AF, IL-22) were evaluated in the serum of control and infected animals at 4, 7, 11, and 14 days post-treatment.

**Figure 8:**
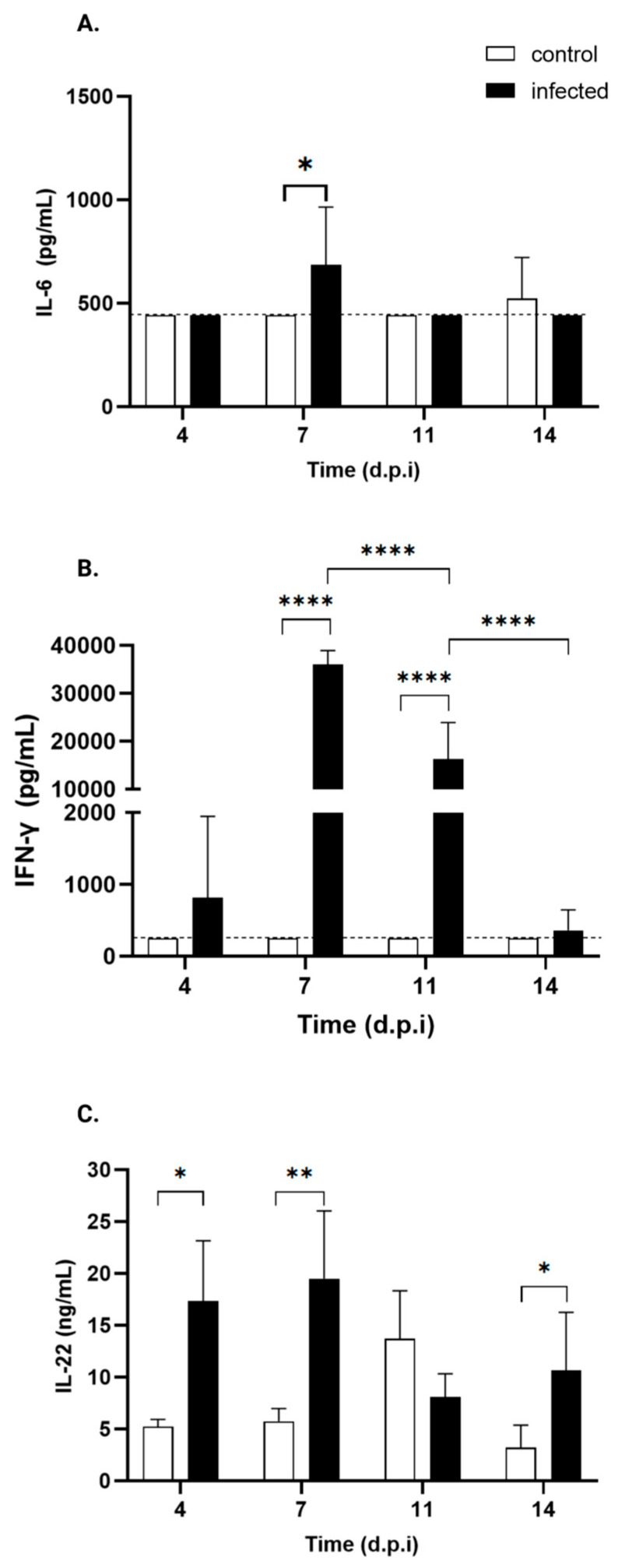
Serum cytokine profiles of control and *T. cruzi* strain Y-infected Swiss mice. ELISA was used to measure serum levels of IL-17 and IL-22, while serum levels of TNF-α, IFN-γ, IL-2, IL-4, IL-5, IL-6, IL-10, and IL-13 were assessed via flow cytometry. Only the cytokines of control (white bars) and *T. cruzi* strain Y-infected Swiss mice (black bars) showing statistically significant differences are presented: (**A**) IL-6, (**B**) IFN-γ, and (**C**) IL-22. * *p* < 0.05, ** *p* < 0.001, **** *p* < 0.0001. Two-tailed ANOVA, followed by Bonferroni’s test, was performed. The dotted line indicates the threshold.

Regarding Th1/Th2 cytokines, IL-6 (Figure 8A) showed a transient, mild but significant 1.5-fold increase at day 7 p.i., while TNF-α, IL-2, and IL-10 serum levels remained undetectable throughout the experiment. However, a mild increase in IFN-γ serum levels was observed in infected mice from day 4 p.i., reaching statistical significance at days 7 and 11 p.i., with levels increasing by 130- and 30-fold, respectively (Figure 8C) (Table 3).

As for Th17 cytokines, infected mice exhibit a significant increase in IL-22 levels (Figure 8C) starting from day 4 p.i., which persists through days 7 and 14 p.i., except for day 11. Specifically, the levels increased by 2.9-fold, 3-fold, and 4.4-fold, respectively. No such changes were observed in IL-17AF.

### 3.6. Organ Macroscopy, Weight, and Parasite Presence

**Figure 9:**
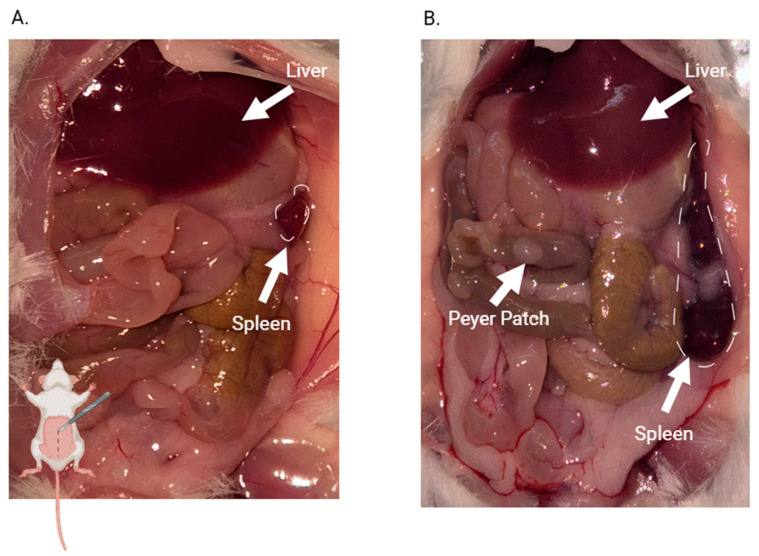
Macroscopic comparison of solid tissues of control and infected mice on day 14 p.i. (**A**) control mouse; (**B**) infected mouse. The mouse drawing in the lower left corner of Figure A indicates which part of the mouse is open in the picture. Arrows point to organs that show morphological or colorimetric characteristics that differ from those in uninfected mice.

Figure 9A shows the organs of an uninfected mouse during necropsy at day 14 p.i. with no evident macroscopic alterations. Both the liver and spleen appear normal in color and size, with all mice exhibiting a similar appearance. In contrast, Figure 9B reveals organomegaly in both the liver and spleen of the infected mice. Both organs show hepatomegaly and splenomegaly. Notably, an increase in esophageal caliber is observed in infected mice, suggestive of megaesophagus.

No macroscopic differences in heart or colon size or morphology are observed between the two groups.

#### 3.6.1. Tissue Weight

Although *T. cruzi* can infect any nucleated cell, specific strains exhibit tropism for certain organs, including the esophagus, liver, spleen, intestine (including the colon), and heart [33]. The relative weights of the esophagus, heart, spleen, and liver were quantified in both infected and control mice throughout the infection process. Tissue weights were normalized to the total body weight to account for natural body weight increase.

**Figure 10:**
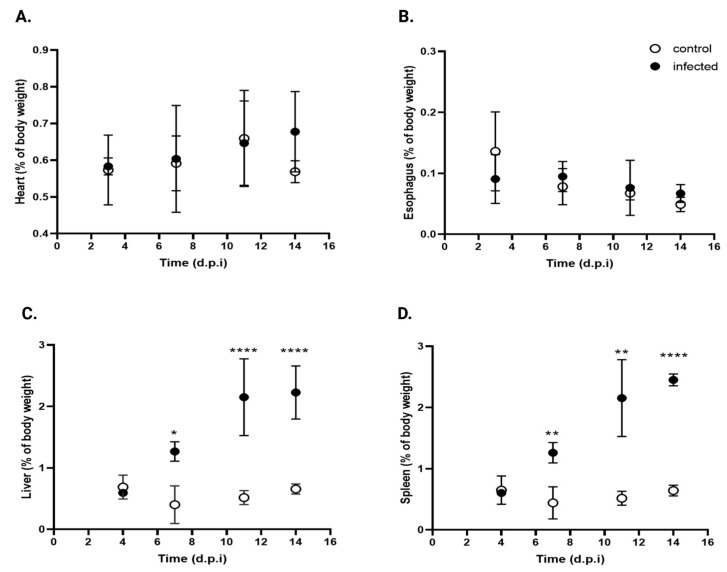
Comparative organ weights in infected and control mice. (**A**) Heart, (**B**) esophagus, (**C**) liver, and (**D**) spleen expressed as percentage of body weight in control (open circles) and *T. cruzi* strain Y-infected mice (black circles). Values are expressed as mean ± standard deviation. Data were analyzed using ANOVA followed by Bonferroni’s two-tailed test. * *p* < 0.05, ** *p* < 0.001, and **** *p*< 0.0001. d.p.i.: days post-infection.

Figure 10 shows the kinetics of organ weight at 4, 7, 11, and 14 d.p.i. Infected mice showed no significant changes in the relative weights of the heart (Figure 10A) or esophagus (Figure 10B), although the esophagus was visibly thicker than in the control mice, suggestive of early megaesophagus.

In contrast, a significant increase in liver weight was noted (Figure 10C), with * 3.5-, **** 4.3-, and **** 3.4-fold increases at the same time points (* *p* < 0.05, **** *p* < 0.001). The spleen (Figure 10D) showed the same trend, with ** 2,8-, ** 4.2-, and **** 3.8-fold increases over control values at 7, 11, and 14 d.p.i., respectively (** *p* < 0.001, **** *p* < 0.0001).

These findings suggest that under the experimental conditions, *T. cruzi* infection selectively induces hepatomegaly and splenomegaly, while the other organs studied remain unchanged.

#### 3.6.2. *T. cruzi* DNA Detection via cPCR

To detect *T. cruzi* in solid tissues, PCR was performed on tissue samples from both healthy and infected animals. Thus, the organs were collected at 4, 7, 11, and 14 d.p.i., and parasite detection was performed via conventional PCR using gDNA extracted from heart, liver, esophagus, spleen, and colon samples. Table 4 shows the positivity of organs to *T. cruzi* DNA amplification as the experiment progressed. Positive controls were included, as were negative PCR products. Data are shown as the number of positive organs over the total analyzed for each group (*n* positive/*n* total), with percentages noted in parentheses.

At 4 d.p.i., 100% of the evaluated animals had already shown esophageal and colon colonization, and this percentage remained unchanged throughout the course of the experiment. A third of the mice tested were positive for *T. cruzi* in the liver from day 4 p.i. onward, and this percentage increased gradually and steadily, reaching 75%, 80%, and 86% at 7, 11, and 14 d.p.i, respectively. The spleen also showed earlier involvement, with 66% positivity at 4 d.p.i., reaching 100% from 7 d.p.i. onward and remaining at this level until the end of this study. Cardiac colonization occurred later, with 75% positivity at 7 d.p.i. and 60% and 85.7% positivity at 11 and 14 d.p.i., respectively.

## 4. Discussion

Reaching consensus on the host response to *T. cruzi* infection remains a major challenge, mainly due to substantial heterogeneity in outcomes driven by both the choice of animal model and, among them, different breeds and strains, as well as the discrete typing unit (DTU) of the infecting parasite [34]. This variability complicates the evaluation of alternative compounds to the two drugs in use for more than 50 years—benznidazole and nifurtimox—since findings are often difficult to extrapolate beyond the specific model in which they were obtained.

In light of these challenges, it is crucial to develop an in vivo experimental model that serves as a reference and can reproduce the most demanding conditions of *T. cruzi* infection, encompassing both the diversity of host responses and variations in parasite virulence. Reflecting recommendations from several drug discovery groups, including those supported by the DNDi, this study utilizes the virulent Y strain (TcII) [34,35,36] in outbred Swiss female mice, which combine greater genetic diversity with a sensitivity profile suitable for evaluating disease progression in a stringent preclinical context [25]. Despite these recommendations, a comprehensive, integrated characterization of the infection’s systemic dynamics remains limited.

In this study, we assessed multiple parameters at various p.i. time points. This broad approach aimed to identify robust and sensitive markers crucial for evaluating promising anti-*T. cruzi* candidate compounds.

Early optimization identified host age and inoculum size as critical determinants of the type of infection, with younger mice exhibiting higher parasite replication due to their immature immune systems. An inoculum of 5 × 10^4^ trypomastigotes produced a pronounced parasitemia around 7–8 d.p.i.

The total area under the parasitemia curve (AUC) confirmed the high replication capacity of *T. cruzi* in the peripheral blood of Swiss mice, reinforcing the suitability of this model for studying the acute phase of infection. The AUC provides a comprehensive measure that integrates both parasite burden and infection duration, making it a sensitive parameter for assessing therapeutic impact in experimental models [32]. Consequently, any changes in the AUC induced via anti-Chagas treatment could provide a robust marker of drug efficacy.

Additionally, the asymmetry in parasitemia kinetics—characterized by a steeper slope in parasite burden between days 0 and 8, followed by a slower decline between days 8 and 17—highlights the dynamic nature of the infection. The sharp increase reflects rapid parasite replication, while the slower decline likely reflects immune responses and parasite migration to tissues. These kinetic changes further emphasize the potential of AUC alteration as a meaningful indicator of treatment efficacy. It is worth noting that strains of the DTU TcI typically exhibit a more gradual but protracted rise in the parasitemia profile [37].

Control and infected mice showed comparable survival rates throughout the experimental period, even with the high parasite burden detected at day 8 p.i., indicating that the initial parasite inoculum produced a non-lethal acute infection. Clinical signs of illness, such as reduced mobility and weakness, appeared later at day 12 p.i., suggesting the occurrence of progressive deterioration associated with the infection. Despite these symptoms, food and fluid intake remained stable and were not significantly affected by infection status. Therefore, these parameters do not appear to be reliable early indicators of infection in this model.

A moderate decrease in body weight was observed by 7 p.i., followed by a slight increase. However, from day 11 p.i. onward, significant organ enlargement (hepato- and splenomegaly) in the infected mice contributed significantly to overall body weight, masking the true extent of weight loss. This suggests that weight gain or stability does not reflect health improvement but rather internal organ changes due to infection.

This organomegaly is consistent with the predominantly reticulotropic nature of the Y strain, which primarily targets the spleen and liver, both of which are rich in the mononuclear phagocyte system. The enlargement of these organs is likely driven by both parasite sequestration/filtration and the activation of immune responses, leading to immune cell recruitment and proliferation. Histological analysis of these organs obtained at 7 and 14 d.p.i. showed increased cellularity and disruption of standard organ architecture compared with uninfected controls (unpublished results).

Serum creatine phosphokinase (CPK) activity is a biomarker of cardiomyocyte injury in Chagas’ cardiomyopathy [38,39,40]. In our study, total CPK levels were below control baseline on days 4, 7, and 14 p.i., with a significant increase only on day 11 p.i., just after the peak of parasitemia.

While other acute infection models report elevated CPK levels indicating myocardial damage, our findings align with those of Mercado et al., who observed low total CPK activity in a model of acute infection with the Tulahuen strain [41,42]. This may be due to the fact that both the Tulahuen (a TcVI DTU) and Y strains [43] are mainly reticulotropic [44], as opposed to other DTUs, such as TcI, which are mainly myotropic [38]. Notably, all hearts from infected animals were positive for *T. cruzi* by day 7 p.i., 4 days before the CPK increase, suggesting that heart colonization precedes detectable cardiomyocyte damage.

Serum ALT levels peaked at 7 d.p.i., suggesting that infected animals experienced acute liver injury at this time point just before the parasitemia peak. This elevation was transient, as values returned to baseline in subsequent days post-infection. In addition, the detection of *T. cruzi* DNA in the liver via cPCR at day 7 p.i. coincided with the peak of ALT. This temporal association is consistent with the parasite’s presence in hepatic tissue and suggests a potential link between early hepatic infection and hepatocellular injury, driven by mononuclear cell infiltration, Kupffer cell activation, and cytokine-mediated inflammation (i.e., IFN-γ and TNF-α). Notably, this time point also corresponded to the peak of parasitemia and the onset of systemic inflammatory responses, suggesting that both direct parasite-mediated damage and immune-driven mechanisms contribute to liver injury. The subsequent decline in ALT might reflect the resolution of the acute injury peak, as well as the rapid recovery of this tissue or parasite persistence in other non-parenchymal liver populations [45]. These observations underscore the value of day 7 p.i. as a critical window for evaluating hepatic function involvement and identifying potential biomarkers responsive to therapeutic intervention in this murine *T. cruzi* infection model. While the Y strain causes a rapid and high elevation of ALT due to its aggressive liver parasitemia, TcI strains such as the Colombian strain show a distinct patter that matches their gentler parasitemia curve [46].

In contrast, LDH levels remained stable throughout the acute phase, indicating that *T. cruzi* infection does not induce widespread tissue injury during this stage, despite the parasites being present in most evaluated organs from the early days of infection.

Many studies have attempted to associate hematological parameters with the parasitological profile and hematological changes related to Chagas disease, with nearly 70% of infection studies reporting increased leukogram values [47].

In our model, total white blood cell counts remained stable, but differential analysis revealed early, transient changes in leukocyte subpopulations. A significant increase in monocytes was observed at days 4 and 5 p.i., indicating the early activation of the monocyte/macrophage lineage consistent with previous reports [48,49]. Granulocyte counts show a trend toward an increase on day 5 p.i., with a clear and significant increase in their percentage. This coincided with the onset of parasitemia, suggesting that the increasing parasitic load triggers these hematological changes, reflecting an early innate immune response. The innate immune system comprises several cell types, including granulocytes, dendritic cells, monocytes/macrophages, and natural killer cells [50,51]. The circulating neutrophil population, typically comprising 10–30% of leukocytes, is essential during the acute phase of infection [52]. In the case of macrophages, these cells play multiple key roles as professional phagocytes, clearing tissues of parasites and apoptotic cells and promoting inflammation, tissue repair [53], and fibrosis [54,55] during *T. cruzi* infection [56]. 

Several studies support the critical role of neutrophils in regulating macrophage responses during parasitic infections, primarily through paracrine mechanisms rather than direct interaction. For instance, Luna-Gómez et al. [57] showed that neutrophil elastase contributes to enhanced macrophage trypanocidal activity in *T. cruzi*-resistant C57BL/6 mice by promoting increased nitric oxide (NO) and TNF-α production.

Neutrophils also contribute to immunity in other parasitic infections, such as tuberculosis [58], leishmaniasis, *Toxoplasma gondii*, *Plasmodium falciparum*, and *Eimeria bovis* tuberculosis [59], through phagocytosis, the production of reactive oxygen/nitrogen species, cytokine release, and the formation of neutrophil extracellular trap (NET) tuberculosis [60].

By day 7 p.i., these changes in monocyte and granulocyte populations had resolved, likely due to rapid tissue infiltration and compensatory bone marrow output. In contrast, lymphocyte counts decreased on day 5 p.i., with a significant decrease in percentage values on days 4 and 5 p.i., before returning to baseline. Lymphocyte depletion during *T. cruzi* infection has been linked to apoptotic mechanisms [13,61], though the precise cause of this transient decrease in our study remains unclear.

No changes in red blood cell count or erythrocyte parameters were observed, contradicting other studies reporting decreases in these parameters in *T. cruzi*-infected mice [62,63,64].

Temporal analysis of platelet counts in *T. cruzi*-infected mice revealed a significant decrease on day 4 p.i., coinciding with the onset of bloodstream parasitemia. This reduction occurred without changes in MPV, suggesting that thrombocytopenia was driven by infection-related mechanisms rather than alterations in thrombopoiesis. Previous studies suggest that *T. cruzi* infection induces platelet clearance through trans-sialidase activity, which cleaves sialic acid residues from the platelet surface, marking them for rapid elimination by hepatic Kupffer cells [65]. Interestingly, from days 8 to 14 p.i., infected animals showed a trend of having lower platelet counts than control mice, with a significant difference observed on day 8. This tendency coincided with a marked increase in serum IFN-γ levels starting at day 7 p.i., consistent with the activation of a Th1-type immune response. IFN-γ has been implicated in transient hematopoietic suppression, including impaired megakaryopoiesis and thrombopoiesis, which could explain the occurrence of sustained reduction in platelet counts despite stable MPV [66]. The kinetics of mixed Th1/Th2 (IFN-γ, TNF-α, IL2, IL-6, and IL-10), strict Th2 (IL-5 and IL-4), and Th17 (IL17/AF, IL-22) cytokines were evaluated in serum at 4, 7, 11, and 14 days p.i.

These findings align with prior evidence that *T. cruzi* infection induces a hypercoagulable state during the acute phase of Chagas disease [67,68]. Although mice may not present apparent clinical symptoms during these early stages, they can already show coagulation alterations that promote thrombus formation, affecting platelet counts [69,70]. Additionally, clinical studies have documented increased platelet adhesion and aggregation, as well as prothrombotic markers, in the early chronic phase of infection [71,72], reinforcing the notion of an infection-driven imbalance in hemostasis.

Regarding the cytokine kinetics of the mixed Th1/Th2 profile (IFN-γ, TNF-α, IL2, IL-6, and IL-10), strict Th2 (IL-5 and IL-4), and Th17 (IL17/AF, IL-22), these molecules were evaluated in the serum of control and infected animals at 4, 7, 11, and 14 days post-treatment.

IL-22 increased at all points, except at 11 d.p.i. As a Th17-associated cytokine with antagonistic–proinflammatory/protective functions [73], IL-22 is known to be induced during *T. cruzi* infection in spleen, heart, and serum in an IL-23-dependent manner in C57Bl/6 mice, contributing to host protection [43,74]. Similar IL-22 profiles occur in protozoan [75], mycobacterial [76], bacterial [77], and viral infections [78].

The protective effect of IL-22 is mediated not only by CD4+ T cells but also by natural killer (NK) cells, primarily the iNKT subset [45]. Thus, both the innate and adaptive immune responses contribute to the mitigation of tissue damage caused by inflammation.

In contrast, IL-17A/F, another Th127-cytokine secreted by CD8+ T cells, γδ T cells, NK, neutrophils and innate lymphoid cells [79,80,81], known to reduce inflammation and mortality during *T. cruzi* infection [82,83], remained undetected throughout the experiment.

IFN-γ showed a mild increase at day 4 p.i., along with significant increases at 7 and 11 d.p.i., (130-fold and 30-fold, respectively). These changes parallelled parasite detection via cPCR at 4 d.p.i. in the colon and esophagus and higher tissue involvement at 7 d.p.i., when the spleen was fully colonized and the heart and liver were more compromised.

Early hepatic changes, including the loss of Kupffer cells and recruitment of other myeloid cells (i.e., monocytes), capable of secreting IFN-γ and TNF-α [62], likely contribute to systemic cytokine elevations. Although *T. cruzi* was detected in the liver of 66% of infected mice as early as 4 d.p.i., supporting the notion that the systemic increase in IFN-γ levels reflects early hepatic immune activation, no significant increase in TNF-α serum levels was observed at any time point. In fact, Th1 cytokines, such as TNF-α and IL-2, together with the regulatory cytokine IL-10 and Th2 cytokines, including IL-4 and IL-5, remained undetectable throughout the experiment. Although we lack direct evidence of in situ cytokine production in the liver, our data suggest that systemic cytokine levels likely reflect the occurrence of early hepatic immune activation in response to infection.

IL-6, a pleiotropic cytokine, showed a mild but significant 1.5-fold increase at 7 d.p.i., consistent with its role in promoting M2 macrophage polarization and regulating nitric oxide through IL-1β inhibition to balance parasite burden and control oxidative stress [84].

The combination of robust IFN-γ induction and a modest increase by 7-11 d.p.i. indicates a predominantly Th1-driven response, promoting the recruitment of monocyte-derived macrophages [85,86]. At the time point at which IFN-γ peaks, granulocyte and monocyte levels increased, with monocyte levels continuing to increase even after granulocyte levels subsided. This pattern supports the previous finding that neutrophils and monocytes are key early controllers of parasite burden [57].

The sustained IFN-γ response until at least day 11 p.i. coincided with parasite clearance from the blood, suggesting the activation of the adaptive response responsible for pathogen elimination.

The immunological profiles observed in this study can be attributed to the reticulotropic nature of the TcII DTU used in this model, and they would likely differ greatly in DTUs with different tissue tropism.

To simplify testing of the relevant markers described here, Table 5 lists the most important days to search for changes in key parameters that could help to guide decisions on GO or NO-GO for generating promising leads.

## 5. Conclusions

New therapeutic interventions are urgently required for Chagas disease. A reliable and standardized in vivo model across drug discovery groups is crucial for the objective comparison of promising molecules across different research groups. This study provides a comprehensive characterization of the *T. cruzi* Y strain in Swiss mice, which reflects consensus-based recommendations from several drug discovery consortia as a benchmark for initial efficacy screening. By integrating parasitological, biochemical, and immunological data, we move beyond isolated metrics to provide a longitudinal map of infection dynamics. While subsequent validation against other DTUs (e.g., TcI) remains essential for confirming broad-spectrum activity, this integrated model provides a detailed benchmark for optimizing experimental design and selecting precise endpoints in preclinical drug discovery.

## Figures and Tables

**Table 1:** Selected primers for conventional PCR of *T. cruzi*.

Primer Name	Sequence ^a^ (5’–3’)	Nucleotide Position ^b^
Cruzi 1 (forward)	ASTCGGCTGATCGTTTTCGA	27–46
Cruzi 2 (reverse)	AATTCCTCCAAGCAGCGGATA	172–192

^a^ S, C/G. ^b^ The nucleotide position in the DNA satellite sequence (Gen Bank Accession No. AY520036).

**Table 2:** Daily water consumption and food intake of mice *.

d.p.i	Water Consumption/Day/Mouse * mL		Food Intake/Day/Mouse * (g)	
	Control (*n* = 6)	Infected (*n* = 9)	Control (*n* = 6)	Infected (*n* = 9)
8	3.5	3.65	2.7	3.2
14	2.5	2.6	4.2	4.4

* Values represent the mean of daily water and food intake per cage, averaged to individual animals. The values measured at day 0 were subtracted from the measurements at days 8 and 14 post-infection. d.p.i.: days post-infection.

**Table 3:** Serum cytokine levels at different days post-infection.

Serum Cytokine Levels									
d.p.i	4		7		11		14		*p* Value
Cytokine	Control (*n* = 3)	Infected (*n* = 3)	Control (*n* = 4)	Infected (*n* = 3)	Control (*n* = 4)	Infected (*n* = 3)	Control (*n* = 3)	Infected (*n* = 5)	Infected vs. Control at Each Time Point
**IL-22**	5.23 ± 0.69	* 11.94 ± 3.68	5.77 ± 1.21	** 16.43 ± 2.71	13.7 ± 4.63	8.12 ± 2.212	3.22 ± 2.16	8.75 ± 6.45	** *p* < 0.001 * *p* < 0.05
**IL-17**	5.85 ± 0.96	4.53 ± 0.81	5.38 ± 1.94	5.24 ± 0.91	7.94 ± 1.84	6.07 ± 0.81	6.04 ± 1.48	6.22 ± 1.47	NS
**TNF-** **α**	<1170	<1170	<1170	<1170	<1170	<1170	<1170	<1170	NS
**INF-** **γ**	<255.4	819 ± 1127	<255.4	**** 36,077 ± 2845	<255.4	* 16,350 ± 7563	<255.4	368.8 ± 284	**** *p* < 0.0001 * *p* < 0.05
**IL-2**	182.19	182.19	182.19	182.19	182.19	182.19	182.19	182.19	NS
**IL-4**	<387.65	<387.65	<387.65	<387.65	<387.65	<387.65	<387.65	<387.65	NS
**IL-5**	<488.5	<488.5	<488.5	<488.5	<488.5	<488.5	<488.5	<488.5	NS
**IL-6**	<442.6	<442.6	<442.6	* 685.2 ± 265.7	<442.6	<442.6	<442.6	523.5 ± 198	* *p* < 0.05
**IL-10**	248.75	248.75	248.75	248.75	248.75	248.75	248.75	248.75	NS
**IL-13**	<595.23	<595.23	<595.23	<595.23	<595.23	<595.23	<595.23	<595.23	NS

*, ** and **** represent significant differences at *p* < 0.05, *p* < 0.001, and *p* < 0.0001, respectively. NS: not significant; <: values below detection threshold. d.p.i.: days post-infection. Numbers in parentheses indicate the number of animals per group.

**Table 4:** Organ-specific detection of *T. cruzi* DNA via PCR at different days post-infection.

*T. cruzi* Positive Organs						
d.p.i	4	7	11	14	Controls	
Tissue	*n* Positive/*n* Total (96)				Negative	Positive
**Esophagus**	3/3 (100)	3/3 (100)	5/5 (100)	7/7 (100)	0/4 (0)	2/2 (100)
**Colon**	3/3 (100)	4/4 (100)	5/5 (100)	7/7 (100)	0/4 (0)	2/2 (100)
**Liver**	2/3 (66)	3/4 (75)	4/5 (80)	6/7 (86)	0/4 (0)	2/2 (100)
**spleen**	2/3(66)	4/4 (100)	5/5(100)	7/7 (100)	0/4 (0)	2/2 (100)
**Heart**	0/3 (0)	3/4(75)	4/5 (60)	6/7 (85.7)	0/4 (0)	2/2 (100)

d.p.i.: days post-infection.

**Table 5:** Prominent time points and biomarkers used to evaluate differences compared to untreated controls.

	Days Post Infection											
	0	4	5	7	8	9	10	11	12	13	14	17
**Parasitemia**			+	+	+	+	+	+				
**Clinical Symptoms**									+	+		
**Weight**				−								
**Functional Markers**												
ALT				+								
CPK		+		+				+			+	
**Hematological markers**												
Absolute monocytes		+	+								+	
Percent Monocytes		+	+									
Percent granulocytes		+										
Percent lymphocytes		−	−									
Platelets		−				−						
Plateletcrit		−			−	−						
**Th1/Th2 cytokines**												
IL-6				+								
IFN-γ				+				+				
Th17 cytokines												
IL-22		+		+							+	
**Organ Necropsis**												
Liver size												+
Esophagus caliber												+
Spleen size												+
**Organ weight**												
Liver		+		+				+			+	
Spleen				+				+			+	
**Organ PCR Positivity %**												
Esophagus		100		100				100			100	
Colon		100		100				100			100	
Spleen		66		100				100			100	
Liver		33		75				80			86	
Heart				75				60			86	

+ = Increase, − = Decrease, Highlighted cells indicate the best days for evaluating the biomarkers of disease.

## Data Availability

The original contributions presented in this study are included in the article/Supplementary Materials. Further inquiries can be directed to the corresponding author.

## Supplementary Materials

The following supporting information can be downloaded at https://www.mdpi.com/article/10.3390/pathogens15010107/s1. Information S1: Optimization of Mouse Age and Inoculum Dose for Quantitative Assessment of Parasitemia. Figure S1: Parasitemia in Swiss mice.

## Abbreviations

The following abbreviations are used in this manuscript:

*T. cruzi*	*Trypanosoma cruzi*
d.p.i.	days post-infection

## References

[1] Lidani K.C.F., Andrade F.A., Bavia L., Damasceno F.S., Beltrame M.H., Messias-Reason I.J., Sandri T.L. (2019). Chagas disease: From discovery to a worldwide health problem. Front. Public Health.

[2] de Araújo C.A., Mayer C., Waniek P.J., Azambuja P., Jansen A.M. (2016). Differentiation of *Trypanosoma cruzi* I (TcI) and *T. cruzi* II (TcII) genotypes using genes encoding serine carboxypeptidases. Parasitol. Res..

[3] Zingales B., Andrade S.G., Briones M.R., Campbell D.A., Chiari E., Fernandes O., Guhl F., Lages-Silva E., Macedo A.M., Machado C.R. (2009). A new consensus for *Trypanosoma cruzi* intraspecific nomenclature: Second revision meeting recommends TcI to TcVI. Mem. Inst. Oswaldo Cruz..

[4] Brenière S.F., Waleckx E., Barnabé C. (2016). Over six thousand *Trypanosoma cruzi* strains classified into discrete typing units (DTUs): Attempt at an inventory. PLoS Negl. Trop. Dis..

[5] Andrade S.G., Figueira R.M., Carvalho M.L., Gorini D.F. (1975). Influência da cepa do *Trypanosoma cruzi* na resposta à terapêutica experimental pelo Bay 2502. Rev. Inst. Med. Trop. São Paulo.

[6] De Castro S.L., De Meirelles M.N. (1987). Effect of drugs on *Trypanosoma cruzi* and on its interaction with heart muscle cell “in vitro”. Mem. Inst. Oswaldo Cruz.

[7] Melo R.C., Brener Z. (1978). Tissue tropism of different *Trypanosoma cruzi* strains. J. Parasitol..

[8] Neal R.A., Van Bueren J. (1988). Comparative studies of drug susceptibility of five strains of *Trypanosoma cruzi* in vivo and in vitro. Trans. R. Soc. Trop. Med. Hyg..

[9] Roval L.E., Aoki A., Gerez de Burgos N.M., Blanco A. (1990). Effect of Gossypol on trypomastigotes and amastigotes of *Trypanosoma cruzi*. J. Protozool..

[10] Shelly E.M., Acuna-Soto R., Ernst K.C., Sterling C.R., Brown H.E. (2016). A critical assessment of officially reported Chagas disease surveillance data in Mexico. Public Health Rep..

[11] World Health Organization Chagas Disease (Also Known as American Trypanosomiasis). https://www.who.int/news-room/fact-sheets/detail/chagas-disease-(american-trypanosomiasis).

[12] Queiroga T.B.D., Pereira N.S., da Silva D.D., Andrade C.M., de Araújo Júnior R.F., Brito C.R.D.N., Galvão L.M.D.C., da Câmara A.C.J., Nascimento M.S.L., Guedes P.M.M. (2021). Virulence of *Trypanosoma cruzi* Strains Is Related to the Differential Expression of Innate Immune Receptors in the Heart. Front. Cell Infect. Microbiol..

[13] Henriques-Pons A., Oliveira G.M., Paiva M.M., Correa A.F., Batista M.M., Bisaggio R.C., Liu C.C., Cotta-De-Almeida V., Coutinho C.M., Persechini P.M. (2002). Evidence for a perforin-mediated mechanism controlling cardiac inflammation in *Trypanosoma cruzi* infection. Int. J. Exp. Pathol..

[14] Osorio L., Ríos I., Gutiérrez B., González J. (2012). Virulence factors of *Trypanosoma cruzi*: Who is who?. Microbes Infect..

[15] Amato Neto V. (2010). Origin of the “Y strain” of *Trypanosoma cruzi*. Rev. Inst. Med. Trop. São Paulo.

[16] Tuttle A.H., Philip V.M., Chesler E.J., Mogil J.S. (2018). Comparing phenotypic variation between inbred and outbred mice. Nat. Methods.

[17] McCormick D.L., Faqi A.S. (2024). Chapter 12—Preclinical Evaluation of Carcinogenicity Using Standard-Bred and Genetically Engineered Rodent Models. A Comprehensive Guide to Toxicology in Nonclinical Drug Development.

[18] Enriquez J., Mims B.M.D., Trasti S., Furr K.L., Grisham M.B. (2020). Genomic, microbial and environmental standardization in animal experimentation limiting immunological discovery. BMC Immunol..

[19] Sunagar R., Kumar S., Namjoshi P., Rosa S.J., Hazlett K.R.O., Gosselin E.J. (2018). Evaluation of an outbred mouse model for Francisella tularensis vaccine development and testing. PLoS ONE.

[20] Martin M.D., Danahy D.B., Hartwig S.M., Harty J.T., Badovinac V.P. (2017). Revealing the Complexity in CD8 T Cell Responses to Infection in Inbred C57B/6 versus Outbred Swiss Mice. Front. Immunol..

[21] Berton R.R., Jensen I.J., Harty J.T., Griffith T.S., Badovinac V.P. (2022). Inflammation Controls Susceptibility of Immune-Experienced Mice to Sepsis. Immunohorizons.

[22] Andrade L.O., Machado C.R., Chiari E., Pena S.D., Macedo A.M. (2002). *Trypanosoma cruzi:* Role of host genetic background in the differential tissue distribution of parasite clonal populations. Exp. Parasitol..

[23] Rice M., O’Brien S. (1980). Genetic variance of laboratory outbred Swiss mice. Nature.

[24] Chia R., Achilli F., Festing M., Fisher E.M.C. (2005). The origins and uses of mouse outbred stocks. Nat. Genet..

[25] Romanha A.J., Castro S.L., Soeiro Mde N., Lannes-Vieira J., Ribeiro I., Talvani A., Bourdin B., Blum B., Olivieri B., Zani C. (2010). In vitro and in vivo experimental models for drug screening and development for Chagas disease. Mem. Inst. Oswaldo Cruz.

[26] Endelman J.B. (2025). Genomic prediction of heterosis, inbreeding control, and mate allocation in outbred diploid and tetraploid populations. Genetics.

[27] Piron M., Fisa R., Casamitjana N., López-Chejade P., Puig L., Vergés M., Gascón J., Gómez i Prat J., Portús M., Sauleda S. (2007). Development of a real-time PCR assay for *Trypanosoma cruzi* detection in blood samples. Acta Trop..

[28] Mariano F.S., Gutierrez F.R., Pavanelli W.R., Milanezi C.M., Cavassani K.A., Moreira A.P., Ferreira B.R., Cunha F.Q., Cardoso C.R., Silva J.S. (2008). The involvement of CD4+CD25+ T cells in the acute phase of *Trypanosoma cruzi* infection. Microbes Infect..

[29] Mateus J., Guerrero P., Lasso P., Cuervo C., González J.M., Puerta C.J., Cuéllar A. (2019). An animal model of acute and chronic Chagas disease with the reticulotropic Y strain of *Trypanosoma cruzi* that depicts the multifunctionality and dysfunctionality of T cells. Front. Immunol..

[30] Martín-Escolano J., Marín C., Rosales M.J., Tsaousis A.D., Medina-Carmona E., Martín-Escolano R. (2022). An updated view of the *Trypanosoma cruzi* life cycle: Intervention points for an effective treatment. ACS Infect. Dis..

[31] Guerra-Slompo E.P., Picchi-Constante G.F.A., Marek M., Romier C., Sippl W., Zanchin N.I.T. (2023). In cellulo and in vivo assays for compound testing against *Trypanosoma cruzi*. STAR Protoc..

[32] Sowunmi A., Gbotosho G.O., Happi C.T., Folarin O., Okuboyejo T., Michael O., Fatunmbi B. (2011). Use of area under the curve to evaluate the effects of antimalarial drugs on malaria-associated anemia after treatment. Am. J. Ther..

[33] Santi-Rocca J., Fernandez-Cortes F., Chillón-Marinas C., González-Rubio M.L., Martin D., Gironès N., Fresno M. (2017). A multi-parametric analysis of *Trypanosoma cruzi* infection: Common pathophysiologic patterns beyond extreme heterogeneity of host responses. Sci. Rep..

[34] Barnabé C., Mobarec H.I., Jurado M.R., Cortez J.A., Brenière S.F. (2016). Reconsideration of the seven discrete typing units within the species *Trypanosoma cruzi*: A new proposal of three reliable mitochondrial clades. Infect. Genet. Evol..

[35] Aleixo D.L., Ferraz F.N., Ferreira E.C., de Lana M., Gomes M.L., de Abreu Filho B.A., de Araújo S.M. (2012). Highly diluted medication reduces parasitemia and improves experimental infection evolution by *Trypanosoma cruzi*. BMC Res. Notes.

[36] Telleria J., Costales J.A. (2025). An overview of *Trypanosoma cruzi* biology through the lens of proteomics: A review. Pathogens.

[37] Barrera Y.K., Guevara J.M., Pavía P.X., Montilla M., Nicholls R.S., Parra E., Puerta C.J. (2008). Evaluation of TcH2AF-R and S35-S36 primers in PCR tests for the detection of *Trypanosoma cruzi* in mouse cardiac tissue. Biomedica.

[38] de Souza W., de Carvalho T.M.U., Telleria J., Tibayrenc M. (2010). Ultrastructure of *Trypanosoma cruzi* and its interaction with host cells. American Trypanosomiasis.

[39] Kroll-Palhares K., Silvério J.C., Silva A.A., Michailowsky V., Marino A.P., Silva N.M., Carvalho C.M., Pinto L.M., Gazzinelli R.T., Lannes-Vieira J. (2008). TNF/TNFR1 signaling up-regulates CCR5 expression by CD8+ T lymphocytes and promotes heart tissue damage during *Trypanosoma cruzi* infection: Beneficial effects of TNF-alpha blockade. Mem. Inst. Oswaldo Cruz.

[40] Carvalho C.M., Silverio J.C., da Silva A.A., Pereira I.R., Coelho J.M., Britto C.C., Moreira O.C., Marchevsky R.S., Xavier S.S., Gazzinelli R.T. (2012). Inducible nitric oxide synthase in heart tissue and nitric oxide in serum of *Trypanosoma cruzi*-infected rhesus monkeys: Association with heart injury. PLoS Negl. Trop. Dis..

[41] Mercado T.I., Garbus J. (1979). Creatine phosphokinase isoenzymes and *Trypanosoma cruzi* infections. Comp. Biochem. Physiol. B.

[42] Erdmann H., Behrends J., Hölscher C. (2016). During acute experimental infection with the reticulotropic *Trypanosoma cruzi* strain Tulahuen IL-22 is induced IL-23-dependently but is dispensable for protection. Sci. Rep..

[43] de Diego J.A., Penin P., del Rey J., Mayer R., Gamallo C. (1991). A comparative pathological study of three strains of *Trypanosoma cruzi* in an experimental model. Histol. Histopathol..

[44] da Cunha G.M.R., Azevedo M.A., Nogueira D.S., de Carvalho Clímaco M., Ayala E.V., Chunga J.A.J., La Valle R.J.Y., da Cunha Galvão L.M., Chiari E., Brito C.R.N. (2021). α-Gal immunization positively impacts *Trypanosoma cruzi* colonization of heart tissue in a mouse model. PLoS Neglected Trop. Dis..

[45] de Lima Pereira Dos Santos C., Vacani-Martins N., Cascabulho C.M., Pereira M.C.S., Crispe I.N., Henriques-Pons A. (2022). In the acute phase of *Trypanosoma cruzi* infection, liver lymphoid and myeloid cells display an ambiguous phenotype combining pro- and anti-inflammatory markers. Front. Immunol..

[46] Duz A.L., Vieira P.M., Roatt B.M., Aguiar-Soares R.D., Cardoso J.M., Oliveira F.C., Reis L.E., Tafuri W.L., Veloso V.M., Reis A.B. (2014). The TcI and TcII *Trypanosoma cruzi* experimental infections induce distinct immune responses and cardiac fibrosis in dogs. Mem. Inst. Oswaldo Cruz.

[47] Villalba-Alemán E., Justinico D.L., Sarandy M.M., Novaes R.D., Freitas M.B., Gonçalves R.V. (2018). Haematological alterations in non-human hosts infected with *Trypanosoma cruzi*: A systematic review. Parasitology.

[48] Calabrese K.S., Lagrange P.H., Da Costa S.C. (1996). Chagas’ disease: Enhancement of systemic inflammatory reaction in cyclophosphamide treated mice. Int. J. Immunopharmacol..

[49] Domingues C.S., Hardoim D.J., Souza C.S., Cardoso F.O., Mendes V.G., Previtalli-Silva H., Abreu-Silva A.L., Pelajo-Machado M., da Costa G.S.C., Calabrese K.S. (2015). Oral Outbreak of Chagas Disease in Santa Catarina, Brazil: Experimental Evaluation of a Patient’s Strain. PLoS ONE.

[50] Brener Z., Gazzinelli R.T. (1997). Immunological control of *Trypanosoma cruzi* infection and pathogenesis of Chagas’ disease. Int. Arch. Allergy Immunol..

[51] Umekita L.F., Mota I. (2000). How are antibodies involved in the protective mechanism of susceptible mice infected with *T. cruzi*?. Braz. J. Med. Biol. Res..

[52] Camargo I.J.B., Araujo P.M.F., Sakurada J.K., Stach-machado D.R., Rancel H.A., Silva P. (1991). *Trypanosoma cruzi*: Early resistance induced by culture derived trypomastigotes. Exp. Parasitol..

[53] Bosurgi L., Cao Y.G., Cabeza-Cabrerizo M., Tucci A., Hughes L.D., Kong Y., Weinstein J.S., Licona-Limon P., Schmid E.T., Pelorosso F. (2017). Macrophage function in tissue repair and remodeling requires IL-4 or IL-13 with apoptotic cells. Science.

[54] Choudhuri S., Garg N.J. (2020). *Trypanosoma cruzi* induces the PARP1/AP-1 pathway for upregulation of metalloproteinases and transforming growth factor beta in macrophages: Role in cardiac fibroblast differentiation and fibrosis in Chagas disease. mBio.

[55] Waghabi M.C., Ferreira R.R., Abreu R.D.S., Degrave W., de Souza E.M., Bailly S., Feige J.J., de Araújo-Jorge T.C. (2022). Transforming growth factor-ß as a therapeutic target for the cardiac damage of Chagas disease. Mem. Inst. Oswaldo Cruz.

[56] Cerbán F.M., Stempin C.C., Volpini X., Carrera Silva E.A., Gea S., Motran C.C. (2020). Signaling pathways that regulate *Trypanosoma cruzi* infection and immune response. Biochim. Biophys. Acta Mol. Basis Dis..

[57] Luna-Gomes T., Filardy A.A., Rocha J.D., Decote-Ricardo D., LaRocque-de-Freitas I.F., Morrot A., Bozza P.T., Castro-Faria-Neto H.C., DosReis G.A., Nunes M.P. (2014). Neutrophils increase or reduce parasite burden in *Trypanosoma cruzi*-infected macrophages, depending on host strain: Role of neutrophil elastase. PLoS ONE.

[58] Fu L.M. (2003). The potential of human neutrophil peptides in tuberculosis therapy. Int. J. Tuberc. Lung Dis..

[59] Ribeiro-Gomes F.L., Otero A.C., Gomes N.A., Moniz-De-Souza M.C., Cysne-Finkelstein L., Arnholdt A.C., Calich V.L., Coutinho S.G., Lopes M.F., DosReis G.A. (2004). Macrophage interactions with neutrophils regulate *Leishmania major* infection. J. Immunol..

[60] Sousa-Rocha D., Thomaz-Tobias M., Diniz L.F., Souza P.S., Pinge-Filho P., Toledo K.A. (2015). *Trypanosoma cruzi* and its soluble antigens induce NET release by stimulating Toll-Like Receptors. PLoS ONE.

[61] Sanoja C., Carbajosa S., Fresno M., Gironès N. (2013). Analysis of the dynamics of infiltrating CD4(+) T cell subsets in the heart during experimental *Trypanosoma cruzi* infection. PLoS ONE.

[62] Malvezi A.D., Cecchini R., de Souza F., Tadokoro C.E., Rizzo L.V., Pinge-Filho P. (2004). Involvement of nitric oxide (NO) and TNF-alpha in the oxidative stress associated with anemia in experimental *Trypanosoma cruzi* infection. FEMS Immunol. Med. Microbiol..

[63] Santos C.D., Levy A.M.A., Toldo M.P.A., Azevedo A.P., Júnior J.C. (2007). Haematological and histopathological findings after ovariectomy in *Trypanosoma cruzi* infected mice. Vet. Parasitol..

[64] Marcondes M.C., Borelli P., Yoshida N., Russo M. (2000). Acute *Trypanosoma cruzi* infection is associated with anemia, thrombocytopenia, leukopenia, and bone marrow hypoplasia: Reversal by nifurtimox treatment. Microbes Infect..

[65] Tribulatti M.V., Mucci J., Van Rooijen N., Leguizamon M.S., Campetella O. (2005). The trans-sialidase from *Trypanosoma cruzi* induces thrombocytopenia during acute Chagas’ disease by reducing the platelet sialic acid contents. Infect. Immun..

[66] de Bruin A.M., Voermans C., Nolte M.A. (2014). Impact of interferon-γ on hematopoiesis. Blood.

[67] Pinazo M.J., Posada Ede J., Izquierdo L., Tassies D., Marques A.F., de Lazzari E., Aldasoro E., Muñoz J., Abras A., Tebar S. (2016). Altered hypercoagulability factors in patients with chronic Chagas disease: Potential biomarkers of therapeutic response. PLoS Negl. Trop. Dis..

[68] Laucella S.A., Segura E.L., Riarte A., Sosa E.S. (1999). Soluble platelet selectin (sP-selectin) and soluble vascular cell adhesion molecule-1 (sVCAM-1) decrease during therapy with benznidazole in children with indeterminate form of Chagas’ disease. Clin. Exp. Immunol..

[69] Choudhuri S., Garg N.J. (2022). Platelets, macrophages, and thromboinflammation in Chagas disease. J. Inflamm. Res..

[70] Tanowitz H.B., Burns E.R., Sinha A.K., Tanowitz H.B. (1990). Enhanced platelet adherence and aggregation in Chagas’ disease: A potential pathogenic mechanism for cardiomyopathy. Am. J. Trop. Med. Hyg..

[71] Herrera R.N., Diaz E., Perez R., Herrera R.N. (2003). Estado protrombótico en estadios tempranos de la enfermedad de Chagas crónica. Rev. Esp. Cardiol..

[72] Herrera R.N., Berman S.G., Luciardi H.L. (2004). Evidence of a prothrombotic state in early stages of chronic Chagas’ disease. Arch. Cardiol. Mex..

[73] Zenewicz L.A., Yancopoulos G.D., Valenzuela D.M., Murphy A.J., Stevens S., Flavell R.A. (2008). Innate and adaptive interleukin-22 protects mice from inflammatory bowel disease. Immunity.

[74] Ferreira B.L., Ferreira É.R., de Brito M.V., Salu B.R., Oliva M.L.V., Mortara R.A., Orikaza C.M. (2018). BALB/c and C57BL/6 mice cytokine responses to *Trypanosoma cruzi* infection are independent of parasite strain infectivity. Front. Microbiol..

[75] Gimblet C., Loesche M.A., Carvalho L., Carvalho E.M., Grice E.A., Artis D., Scott P. (2015). IL-22 protects against tissue damage during cutaneous leishmaniasis. PLoS ONE.

[76] Dhiman R., Indramohan M., Barnes P.F., Nayak R.C., Paidipally P., Rao L.V., Vankayalapati R. (2009). IL-22 produced by human NK cells inhibits growth of Mycobacterium tuberculosis by enhancing phagolysosomal fusion. J. Immunol..

[77] Basu R., O’Quinn Darrell B., Silberger D.J., Schoeb T.R., Fouser L., Ouyang W. (2012). Th22 cells are an important source of IL-22 for host protection against enteropathogenic bacteria. Immunity.

[78] Radaeva S., Sun R., Pan H.N., Hong F., Gao B. (2004). Interleukin 22 (IL-22) plays a protective role in T cell-mediated murine hepatitis: IL-22 is a survival factor for hepatocytes via STAT3 activation. Hepatology.

[79] Cua D.J., Tato C.M. (2010). Innate IL-17-producing cells: The sentinels of the immune system. Nat. Rev. Immunol..

[80] Sutton C.E., Mielke L.A., Mills K.H. (2012). IL-17-producing gammadelta T cells and innate lymphoid cells. Eur. J. Immunol..

[81] Li L., Huang L., Vergis A.L., Ye H., Bajwa A., Narayan V., Strieter R.M., Rosin D.L., Okusa M.D. (2010). IL-17 produced by neutrophils regulates IFN-gamma-mediated neutrophil migration in mouse kidney ischemia-reperfusion injury. J. Clin. Investig..

[82] Boari J.T., Amezcua Vesely M.C., Bermejo D.A., Ramello M.C., Montes C.L., Cejas H., Gruppi A., Acosta Rodríguez E.V. (2012). IL-17RA signaling reduces inflammation and mortality during *Trypanosoma cruzi* infection by recruiting suppressive IL-10-producing neutrophils. PLoS Pathog..

[83] Vesely A.M.C., Rodríguez C., Gruppi A., Acosta Rodríguez E.V. (2020). Interleukin-17 mediated immunity during infections with *Trypanosoma cruzi* and other protozoans. Biochim. Biophys. Acta Mol. Basis Dis..

[84] Sanmarco L.M., Ponce N.E., Visconti L.M., Eberhardt N., Theumer M.G., Minguez Á.R., Aoki M.P. (2017). IL-6 promotes M2 macrophage polarization by modulating purinergic signaling and regulates the lethal release of nitric oxide during *Trypanosoma cruzi* infection. Biochim. Biophys. Acta Mol. Basis Dis..

[85] Gazzinelli R.T., Oswald I.P., Hieny S., James S.L., Sher A. (1992). The microbicidal activity of interferon-gamma-treated macrophages against *Trypanosoma cruzi* involves an L-arginine-dependent, nitrogen oxide-mediated mechanism inhibitable by interleukin-10 and transforming growth factor-beta. Eur. J. Immunol..

[86] Vespa G.N., Cunha F.Q., Silva J.S. (1994). Nitric oxide is involved in control of *Trypanosoma cruzi*-induced parasitemia and directly kills the parasite in vitro. Infect. Immun..

