# Supplementary material for: Kinetics of Biomarkers for Therapeutic Assessment in Swiss Mice Infected with a Virulent Trypanosoma cruzi Strain
Source: Pathogens. 2026 Jan 19;15(1):107. doi: 10.3390/pathogens15010107 (PMC12845268; doi:10.3390/pathogens15010107)
Supplement: Supplementary file 1 [file pathogens-15-00107-s001.zip › pathogens-4031859-supplementary.docx]

**Supplementary Information**

**Kinetics of Biomarkers for Therapeutic Assessment in Swiss Mice Infected with a Virulent *Trypanosoma cruzi* Strain**

María Fernanda Alves-Rosa ^1,2,†^, Doriana Dorta ^1,†^, Alexa Prescilla-Ledezma ^2,3^, Jafeth Carrasco ^1,4^,
Leighanne Bonner ^1,5^, Jon J. Tamayo ^1,5^, Michelle G. Ng ^1^, Adelenis Vega ^1,6^, Melany Morales ^1,6^, Davis Beltran ^7^,
Rosa De Jesús ^2,8^ and Carmenza Spadafora ^1,2,^*

^1^ Division of Human Health and Diseases, Instituto de Investigaciones Científicas y Servicios de Alta
Tecnología (INDICASAT AIP), Panama 0843, Panama; mariaalves@indicasat.org.pa (M.F.A.-R.);
dorianady@gmail.com (D.D.); jafeth_abdul.carrasco@med.lu.se or jcarrasco@indicasat.org.pa (J.C.);
lianmarie26@gmail.com (L.B.); jonj.tamayo23@gmail.com (J.J.T.);
mng@indicasat.org.pa (M.G.N.); adelenis.vega03@gmail.com (A.V.); melanymorales757@gmail.com (M.M.)

^2^ Sistema Nacional de Investigación (SNI), Secretaría Nacional de Ciencia Tecnología e Innovación
(SENACYT), Panama 0843, Panama; alexa_prescilla@yahoo.es (A.P.-L.); rdejesus@indicasat.org.pa (R.D.J.)

^3^ Facultad de Medicina, Departamento de Microbiología Humana, Universidad de Panamá,
Panama 0801, Panama

^4^ Medical Microspectroscopy Research Group, Department of Experimental Medical Science,
Faculty of Medicine, Lund University, 22184 Lund, Sweden

^5^ Escuela de Biotecnología, Facultad de Ciencias de la Salud, Universidad Latina de Panamá,
Panama 0823, Panama

^6^ Facultad de Ciencias Naturales, Exactas y Tecnología, Departamento de Genética y Biología Molecular, Universidad de Panamá, Panama 0801, Panama

^7^ Flow Cytometry Core, GMIHS, Gorgas Memorial Institute of Health Sciences, Panama 0816, Panama;
dbeltran@gorgas.gob.pa

^8^ Bioterio, Instituto de Investigaciones Científicas y Servicios de Alta Tecnología (INDICASAT AIP),
Panama 0843, Panama

***** Correspondence: cspadafora@indicasat.org.pa

^†^ These authors contributed equally to this work.

**Information S1. Optimization of Mouse Age and Inoculum Dose for Quantitative Assessment of Parasitemia**

To determine the optimal parasite inoculum and the age of the mice for assessing *T. cruzi* infection in peripheral blood, four mice were used. Two 4-week-old mice were intraperitoneally inoculated with 200 μl of either 1×10⁴ or 5×10⁴ parasites derived from *in vitro* culture. Another two 8-week-old mice were treated equally. All mice were subjected to periodic blood sampling for quantification of parasites.

As shown in Supplementary Figure 1, parasites were detected in the blood of 4-week-old mice on day 4 post-infection (p.i.), with a peak on day 8. In this group, animals inoculated with the higher parasite load exhibited a maximum parasitemia of approximately 7 × 10^5^ parasites/mL, whereas those receiving the lower inoculum exhibited a significantly lower peak of 5 × 10^5^ parasites/mL on the same day. After day 8, the parasitemia gradually declined. The 8-week-old mice showed a delayed onset of detectable parasitemia, first appearing on day 6 p.i. Of these, mice inoculated with 5 × 10⁴ parasites reached a peak of about 2 × 10⁴/mL, followed by a rapid decrease by day 10. Meanwhile, those inoculated with 1 × 10⁴ parasites showed only a mild increase in parasitemia (1 × 10⁴ parasites/mL) by day 10, at which point the experiment was concluded. Based on these findings, all following experiments were conducted in 4-week-old mice inoculated with 5 × 10⁴ parasites.

**
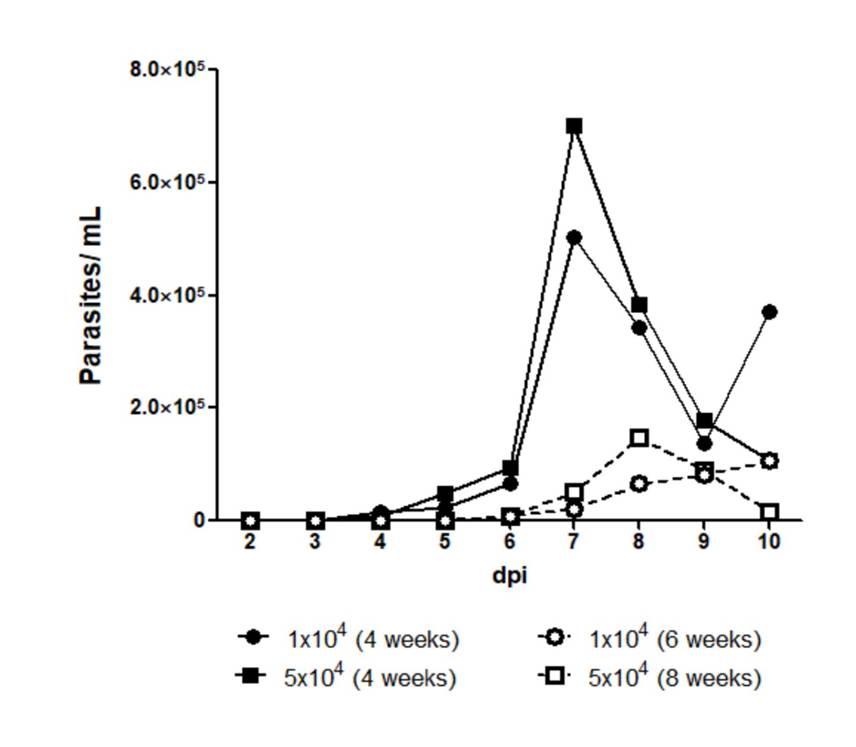
**

**Figure S1. Parasitemia in Swiss mice.** Different-aged mice were infected with *T. cruzi* Y. Parasitemia is expressed as parasites/mL. dpi: days post-infection. n=1 per condition.
